# Driving, Social Distancing, Protective, and Coping Behaviors of Older Adults Before and During COVID-19

**DOI:** 10.1177/07334648221093851

**Published:** 2022-05-11

**Authors:** Catherine M. Roe, Sayeh Bayet, Jamie Hicks, Ann M Johnson, Samantha Murphy, Jason M. Doherty, Ganesh M. Babulal

**Affiliations:** 1 Roe Research LLC, St. Louis, MO, USA; 2 Department of Biomedical Engineering, University of Calgary, Calgary, AB, Canada; 3 Department of Geomatics Engineering, University of Calgary, Calgary, AB, Canada; 4 Department of Neurology, Washington University School of Medicine, St. Louis, MO, USA; 5 Center for Clinical Studies, Washington University School of Medicine, St. Louis, MO, USA; 6 Charles F. and Joanne Knight Alzheimer’s Disease Research Center, Washington University School of Medicine, St. Louis, MO, USA; 7 Department of Psychology, Faculty of Humanities, University of Johannesburg, South Africa

**Keywords:** automobile driving, motor vehicles, COVID-19, pandemic

## Abstract

A thorough understanding of individual characteristics of older adults during the COVID-19 pandemic is critical for managing the ongoing pandemic course and planning for the future pandemics. Here, we explore the impact of the COVID-19 pandemic on driving, social distancing, protective, and coping behaviors of older adults. This study reports data on participants aged above 65 whose driving behaviors are being monitored using Global Positioning System (GPS) devices. Participants completed a COVID-19 survey in May 2020. We found that older adults decreased their number of days driving, number of trips per day, as well as average driving speed, and had fewer speeding incidents following COVID-19 onset. We also show that female and African American older adults engaged in more positive coping and cleaning behaviors, and had greater decreases in the number of days driving during the pandemic. The findings highlight the importance of considering older adults’ individual characteristics for an equitable response to the COVID-19 pandemic.

## Introduction

This project documents daily behaviors of a sample of US older adults before and during the COVID-19 pandemic, as well as their COVID-related beliefs and feelings. The first documented cases of COVID-19 in the US occurred in January 2020 and the disease spread rapidly in the following months (https://www.ajmc.com/view/a-timeline-of-covid19-developments-in-2020). In early March 2020, the danger of the virus became increasingly clear, as growing numbers of people became hospitalized and died. Older adults were found to be especially vulnerable to developing severe symptoms of COVID-19 (https://www.cdc.gov/coronavirus/2019-ncov/need-extra-precautions/older-adults.html), with individuals aged 65+ years being 35 to 80 times more likely to be hospitalized, and between 1100 and 7900 times more likely to die, depending on their specific age, compared to children aged 5 to 17 years (the age group with the lowest morbidity and mortality rates (Centers for Disease Control and Prevention, 2021). As of October 2020, adults 50 years of age and above accounted for 95% of all COVID-19 related deaths in the US (Nania, 2020). Moreover, older adult survivors may be more likely than younger persons to experience post-COVID-19 sequela, with adults aged 60 years or above comprising 51% \of those who developed altered mental status, and 82% of cerebrovascular events (Varatharaj et al., 2020).

With no vaccine available in the US until December of 2020, efforts to fight the virus during 2020 consisted primarily of preventing person-to-person infection by social distancing and wearing protective gear such as masks. Likewise, cleaning and disinfecting measures were used to decrease the likelihood of surface-to-person transmission. Given the central role of individual action in preventing and managing the 2020 spread of COVID-19, assessment of human behavior during this time, especially among the most vulnerable, may provide important insights for managing the ongoing COVID-19 pandemic course and combating future pandemics.

Our longitudinal cohort of study participants, aged 65 years and older, are assessed yearly for the presence and severity of dementia, and additional clinical, cognitive, and driving data are gathered. Beginning in 2017, almost all recruited participants have a data logger installed in their personal vehicle which tracks their latitude, longitude, and speed every 30 seconds while they are driving in their own environments, when and where they choose. Important discrete driving events (e.g., hard braking, impacts) are captured anytime they occur.

Driving reflects valuable information about human behavior over and above that related to the act of driving itself, including the degree to which people reduce the number and places they visit during a pandemic. Although studies using cell phone and other modes to track human movement yield valuable information, they typically reflect human travel on an aggregate basis, without studying the specific movement patterns of an individual across time. In addition, this aggregate data cannot be linked to other information specific to the individual, such as their beliefs about COVID-19 and the extent to which they take part in other pandemic-related behaviors such as handwashing or wearing masks.

Here, we report the results of a natural experiment comparing the driving behavior of older adults before and after the onset of COVID-19 during 2020, and their driving behavior during 2019, the year before the pandemic. Because we had complete, continuously collected driving behavior on participants over this 2-year period, each individual serves as his or her own control, such that any observed changes in driving behavior cannot be attributed to differences in members of the sample over time. Moreover, comparison of 2020 and 2019 behavior accounts for seasonal effects in driving and travel, (Smith et al., 2016; Weast, 2018) which otherwise might be attributed to COVID-19. In May 2020, participants with complete driving behavior 2019–2020, as well as those in our larger longitudinal studies without complete driving data, took part in a survey capturing other behaviors, beliefs, and feelings related to the pandemic.

## Materials and Methods

All study protocols, consent documents, and questionnaires were approved by Washington University Institutional Review Board.

### Participant Sample

Participants were recruited from the community and from the Washington University Knight Alzheimer Disease Research Center and were enrolled in longitudinal studies examining driving behavior in preclinical Alzheimer disease. All participants provided a signed informed consent. Inclusion criteria at baseline were cognitive normality (Clinical Dementia Rating [CDR]) rating of “0,” (Morris, 1993) 65 years of age or above, having a valid driver’s license, driving at least once per week, and meeting minimal visual acuity for state driving guidelines. Washington University Human Research Protection Office approved study protocols and questionnaires (no. 201412024). Based on each participant’s zip code, an updated Social Deprivation Index (SDI) (Butler et al., 2013) (https://www.graham-center.org/rgc/maps-data-tools/sdi/social-deprivation-index.html) was assigned.

### Clinical and Cognitive Assessments

A CDR reflects the presence and severity of dementia and is derived by experienced clinicians who synthesize information obtained from semi-structured interviews with the participant and a collateral source (usually a spouse, friend, or adult child) who knows the participant well. The clinician’s judgment about the presence of dementia was determined using a standard scoring system of 0, reflecting cognitive normality, to 3, signifying severe dementia (Morris, 1993). Participants also completed an annual assessment that included a physical, psychological, neurological, and health examination, as well as the Driving Habits Questionnaire (DHQ) (Owsley et al., 1999).

### Survey

Most active participants in our study cohort were emailed a survey link (developed using Qualtrics) in early May of 2020 and were asked to complete the survey online within 7 days of the original email. They received an emailed reminder to complete the survey 3 days prior to the due date it had not already been returned. If the survey had not been returned by the due date, the participant was contacted to inquire whether they had received the link, and if so, whether they would like to complete the survey. Participants were given the choice of responding to the survey via phone or by the online survey link. For those without an email address (*n* = 10), the participant was called and asked if he/she would be willing to complete the questionnaire over the phone.

The survey used modified questions from the Center for Disease Control and Prevention (CDC) COVID-19 Com-munity Survey Question Bank (https://cde.nlm.nih.gov/formView?tinyId=Kcceysolt) (Version as of April 30, 2020). Survey questions included asking participants about their overall experiences during the pandemic such as places visited outside the home, employment/financial status, use of masks, and overall feelings surrounding the pandemic.

Six subscales were created based on survey responses: Social Distancing, Masking, Cleaning, Positive Coping, Negative Coping, and Maladaptive Behaviors (Supplementary Data 1). For the Masking subscale, persons who endorsed any of the items tended to endorse them all, so this subscale was dichotomized to indicate Any vs. No Masking. The remaining five subscales were continuous and were constructed by adding together the number of items endorsed.

### Driving

Participants’ everyday driving behavior was captured using the Driving Real World In-Vehicle Evaluation System (DRIVES). The DRIVES includes a commercial GPS data logger (G2 Tracking Device, Azuga Inc, San Jose, CA, USA) plugged into the vehicle’s onboard diagnostics-II (OBD-II) port and powered by the vehicle’s battery (Ganesh M. Babulal et al., 2016a, 2016b; Babulal et al., 2017; Roe et al., 2019). The data logger (“chip”) installation requires a vehicle to have been manufactured in 1996 or later, as vehicles made in earlier years were not equipped with an OBD-II port. From ignition-on to ignition-off, the chip samples driving activity every 30 seconds capturing the vehicle’s location (latitude and longitude), speed, date, and time. Additionally, distinct driving events such as hard braking, sudden acceleration, and impacts are captured whenever they occur. The data are collected and transmitted secured servers and downloaded to our lab daily. Participants were given a Bluetooth Low Energy (BLE) beacon, the size and weight of a credit card, that may be placed in a wallet or purse for driver identification. The BLE beacon automatically pairs with the GPS devices when the participant is in the driver’s seat to ensure the participant is driving the car. Variables obtained from the data included number of trips, average miles per trip, average speed, proportion of days with driving, number of trips with hard braking, sudden acceleration, speeding, and percentage of impact.

A trip destination was detected when a change of engine status between on and off was reported. To infer the place type associated with each trip destination, geographic data on points-of-interest (POIs) were extracted from OpenStreetMap (OSM) (OpenStreetMap contributors, 2021). A POI was assigned to a trip destination if the location had the shortest distance to the trip destination and its distance was less than 0.06 Mi. If no POI was in the 0.06 Mi radius of the trip destination, no place type was mapped to the trip destination. The threshold of 0.06 Mi distance between a trip destination and a POI was selected to ensure correct POI mapping (Bohte & Maat, 2009; Nguyen et al., 2020).

Each POI in the OSM database has an associated area layer that identifies a place type. Using the mapping between POIs and place types (Supplementary Table 1), trip destinations are placed into the following 11 categories: place of worship, restaurant, education, leisure, health, food shopping, other shopping, public, money, accommodation, and tourism.

### Statistics

The “entire” sample refers to all participants who responded to the survey. The subsample of participants who also had complete chip data for the entire 1/1/2019–12/31/2020 period is referred to as the “driving” sample.

For the cross-sectional survey data, logistic regression was used to examine associations between demographic (age, gender, African American [AA] vs. White race, education, living alone, SDI), belief and feeling variables and Masking (Any vs. None); and General Linear Models (GLM) examined these associations with the remaining five subscales. Since all independent variables were entered into the models simultaneously, each independent variable was adjusted for the effects of all others.

**Figure 1.**
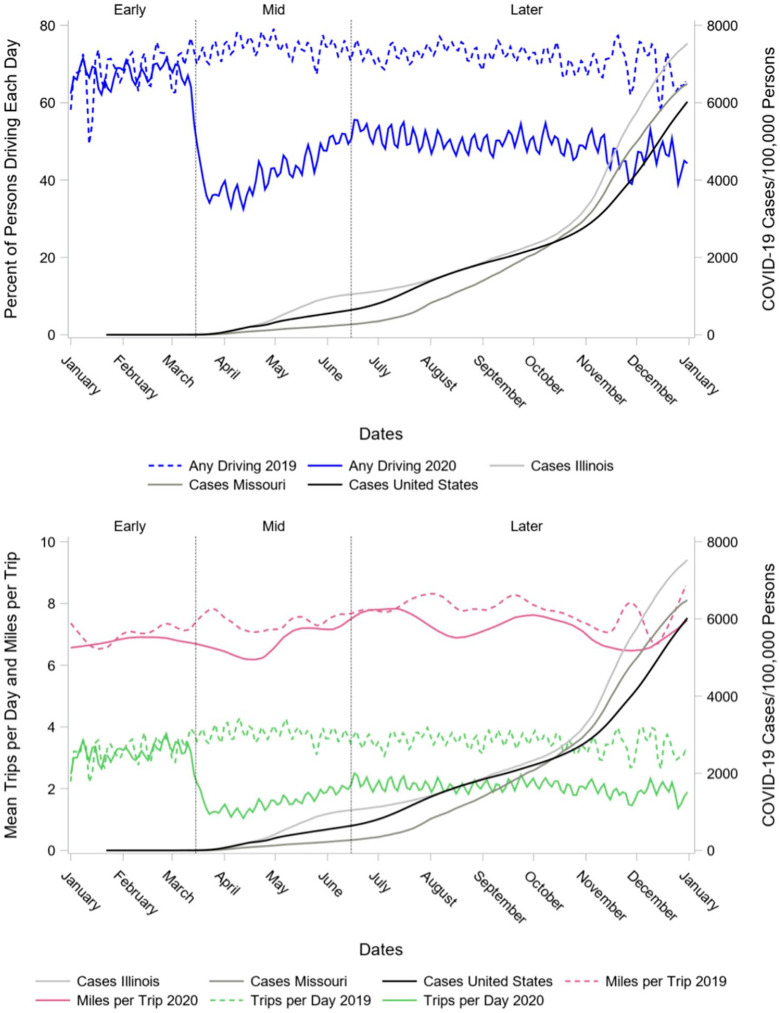
Changes in driving behaviors during and after the rapid acceleration of US COVID-19 cases (**a**) Changes in percentage of people driving each day and (**b**) Changes in number of trips and miles per trip.

Longitudinal changes in driving behavior were first examined by graphing the data. Lowess curves were used to show the mean percentage of persons driving at least once each day over the 2-year period. We divided each year into three epochs based on the changing proportion of persons driving each day during 2020 (Figure 1): Early (January 1–March 14), Mid (March 15–June 14), and Later (June 15–December 31). The end date of March 14 for the Early epoch is the same as that used in a report on driving changes as they occurred at the beginning of the pandemic (Roe et al., 2021). For each participant, we calculated the means on each of the driving and places-traveled variables for each of these six periods.

To examine overall driving changes linked to the pandemic, GLM was used to test for significant differences in Year (2019 vs. 2020), Time (Early, Mid, and Late), and the Year X Time interaction for each driving variable. Year and Time were treated as within-subjects variables, and planned comparisons between 2019 and 2020 for the Early, Mid, and Late time periods were also conducted.

Data from only 2020 were used to test for changes in each of the driving variables over the Early, Mid, and Late epochs as they related to survey-reported demographics, beliefs, and feelings (i.e., the “participant” variables). GLMs were conducted for each driving variable that showed a significant Year X Time interaction in the previous analyses. These GLMs included all participant variables as between-subjects factors, Time as a within-subjects factor, and tested for 2-factor interactions between the participant variables and Time. For example, a significant participant variable (e.g., Gender) X Time interaction would indicate that there was a difference between the genders in the change in the driving variable across 2020). Planned comparisons tested whether there were significant differences across levels of the participant variables within a time epoch. Similar analyses were conducted to determine whether participant variables were associated with differential change in places traveled during 2020.

Unless otherwise noted, for categorical independent variables, numbers reflect least square means ± standard error and the parameter estimate (b) ± standard error for continuous independent variables, from the General Linear Models (GLM).

## Results

Of the 296 active study participants, 292 individuals responded to our survey (98.65% response rate; two participants declined and two could not be contacted). Of these, 180 completed the survey online through Qualtrics (61.64%) and 112 completed the survey over the telephone (38.36%). Data from one participant who quit the survey after answering only a few demographic questions were not included in analyses. From the entire sample of *N* = 291 (age range=61.8–92.6 years), 199 participants had their driving continuously monitored over the period 1/1/2019–12/31/2020 and comprised the driving subsample (Table 1). In the entire sample, the participants had a mean age of 75.4 years (SD 5.0) and 16.5 (SD 2.5) years of education. In addition, approximately half of the participants were women (49%) and only 14% were African American. Only one individual reported that they had tested positive for COVID-19 as of the time of the survey.

**Table 1.** Demographics for all participants and the subsample with driving data available 1/1/19–12/31/20.

Demographics	All (*N* = 291)	Driving subsample (*N* = 199)
Age, mean (SD), y	75.4 (5.0)	74.7 (4.9)
Women, No. (%)	143 (49.1)	77 (46.1)
Education, mean (SD), y	16.5 (2.5)	16.7 (2.3)
African American Race, No. (%)	40 (13.8)	18 (10.8)
Employment Status		
Employed	34 (11.7)	20 (12.3)
Unemployed	1 (0.3)	1 (0.6)
Unable to work	1 (0.3)	0 (0.0)
Homemaker	4 (1.4)	1 (0.6)
Retired	251 (86.3)	141 (86.5)
State of Residence		
Missouri, No. (%)	243 (87.4)	148 (88.6)
Illinois, No. (%)	30 (10.8)	18 (10.8)
Colorado, No. (%)	2 (0.7)	0 (0.0)
Florida, No. (%)	2 (0.7)	0 (0.0)
Tennessee, No. (%)	1 (0.4)	1 (0.4)

### Survey

Women reported more cleaning (3.8 ± 0.1 vs. 3.6 ± 0.2, *p* = .04) and positive coping behaviors (3.8 ± 0.3 vs. 3.4 ± 0.3, *p* = .03) than men, and African Americans reported more of these behaviors than Whites (cleaning=4.0 ± 0.2 vs. 3.4 ± 0.1, *p* < .001; positive coping=3.9 ± 0.3 vs. 3.3 ± 0.2, *p* = .006). African Americans were also more likely to wear masks (odds ratio [OR], 95% confidence interval [CI]=6.26[1.57–25.00], *p* = .009), as were individuals with any of 10 medical risk factors (asthma, chronic lung disease [e.g., emphysema], chronic heart condition, diabetes, chronic kidney disease or dialysis treatment, chronic liver disease, hemoglobin disorders, cancer in the past year, immunosuppressive condition, and severe obesity) for COVID-19 (OR=2.23, 95%CI=1.15–4.31, *p* = .02), and those who showed greater agreement with the survey item, “I believe that by practicing good social distancing behaviors, I can protect myself from COVID-19” (OR=1.49, 95%CI=1.09–2.04, *p* = .01). Individuals who lived alone (OR=0.42, 95%CI=0.21–0.82, *p* = .01) were less likely to wear masks. In addition to being more likely to wear masks (OR=1.49, 95%CI=1.09–2.04, *p* = .01), individuals who believed that they could protect themselves from COVID-19 with social distancing also engaged in more cleaning (b=0.180 ± 0.057, *p* = .002) and positive coping behaviors (b=0.256 ± 0.098, *p* = .009).

Of all independent variables examined, social distancing behavior was only associated with stress due to social distancing, such that individuals who reported more social distancing also reported more stress due to that behavior (b=0.160 ± 0.082, *p* = .05). Greater stress related to social distancing was also related to more cleaning (b=0.139 ± 0.056, *p* = .01) and more negative coping (b=0.204 ± 0.058, *p* < .001) behavior. Older adults who lived in zip codes with higher SDIs also reported more negative coping (b=0.005±0.002, *p* = .04), and individuals who lived alone (9.3 ± 0.44 vs. 8.12 ± 0.44, *p* = .001) and had more years of education (b=0.125 ± 0.064, *p* = .05) took part in more maladaptive behaviors.

### Changes in Naturalistic Driving Behaviors

Significant interactions of Year X Time, indicating that there was a change in driving behavior coincident with the COVID-19 pandemic, were found for five of the eight driving behaviors studied. As shown in Table 2, driving behavior declined disproportionately across 2020 compared to 2019 for any driving, trips/day, miles/trip, speed/trip, and overspeeding. Planned comparisons (Table 2) were used to describe the form of these interactions and indicated that no 2019–2020 differences in these driving behaviors were found for the Early period of the year, but all five showed differences during the Mid portion of the years, as the number of COVID-19 cases in the US began to accelerate more rapidly and additional COVID-19 related mandates and travel restrictions were placed. Differences in any driving, trips/day, and overspeeding persisted into the late part of the years. Although the proportion of trips with impacts was higher for all three time periods during 2019 compared to 2020, the greatest absolute difference between the years occurred during the Mid portion of each year, resulting in a significant Year X Time interaction (Table 2).

**Table 2.** Means and standard deviations (SD) of each of the driving and places-traveled variables for each of six periods of Early (January 1–March 14), Mid (March 15–June 14), and Later (June 15–December 31) portions of 2019 and 2020.

		2019			2020			*p*-values			
Naturalistic driving data	N	Early	Middle	Late	Early	Middle	Late	Year X Time Interaction	Early 2019 vs. 2020	Mid-2019 vs. 2020	Late 2019 vs. 2020
Any driving, mean (SD), proportion of days	199	0.700 (0.215)	0.757 (0.190)	0.743 (0.196)	0.696 (0.251)	0.435 (0.290)	0.500 (0.311)	<.0001	0.7893	<.0001	<.0001
Trips per day, mean (SD), number of trips	199	3.3 (1.7)	3.8 (1.7)	3.6 (1.5)	3.3 (1.9)	1.6 (1.4)	2.1 (1.6)	<.0001	0.7023	<.0001	<.0001
Mean miles per trip, mean (SD), miles	167	25.4 (5.1)	25.7 (4.7)	25.6 (5.0)	25.5 (5.1)	24.6 (5.3)	25.3 (4.7)	0.045	0.8878	0.0046	0.0559
Mean speed per trip, mean (SD), mph	167	25.4 (5.0)	25.7 (4.7)	25.6 (5.0)	25.5 (5.1)	24.6 (5.3)	25.3 (4.7)	0.0021	0.7356	0.0012	0.1636
Mean hard braking, mean (SD), # of brakes	167	0.027 (0.037)	0.025 (0.028)	0.023 (0.027)	0.029 (0.047)	0.026 (0.042)	0.025 (0.041)	0.9642			
Mean hard accelerations, mean (SD), # of acceleration	167	0.011 (0.015)	0.011 (0.017)	0.011 (0.016)	0.015 (0.049)	0.010 (0.022)	0.011 (0.019)	0.1783			
Mean overspeeding, mean (SD), # of speeding events	167	0.017 (0.022)	0.017 (0.021)	0.014 (0.020)	0.012 (0.017)	0.006 (0.011)	0.007 (0.013)	<.0001	<.0001	<.0001	<.0001
Percentage of Impact, mean (SD), % of Impact	167	0.092 (0.501)	0.164 (0.673)	0.177 (0.638)	0.495 (1.684)	0.688 (2.103)	0.562 (1.774)	0.3383			
**Places driven**											
Accommodations, mean (SD), proportion of trips	166	0.018 (0.085)	0.030 (0.118)	0.021 (0.051)	0.020 (0.067)	0.017 (0.095)	0.032 (0.087)	0.175			
Education, mean (SD), proportion of trips	166	0.026 (0.095)	0.021 (0.072)	0.021 (0.070)	0.023 (0.078)	0.012 (0.094)	0.026 (0.106)	0.4654			
Food, mean (SD), proportion of trips	166	0.084 (0.145)	0.080 (0.113)	0.087 (0.135)	0.078 (0.133)	0.120 (0.192)	0.130 (0.193)	0.0012	0.5636	0.008	0.0014
Health, mean (SD), proportion of trips	166	0.090 (0.170)	0.017 (0.128)	0.075 (0.123)	0.078 (0.147)	0.097 (0.190)	0.084 (0.132)	0.1101			
Leisure, mean (SD), proportion of trips	166	0.069 (0.180)	0.081 (0.168)	0.080 (0.161)	0.078 (0.177)	0.051 (0.148)	0.080 (0.159)	0.0847			
Money, mean (SD), proportion of trips	166	0.044 (0.101)	0.040 (0.088)	0.046 (0.101)	0.039 (0.094)	0.030 (0.116)	0.027 (0.058)	0.5378			
Public, mean (SD), proportion of trips	166	0.094 (0.138)	0.093 (0.124)	0.118 (0.142)	0.113 (0.174)	0.099 (0.202)	0.117 (0.168)	0.5647			
Restaurant, mean (SD), proportion of trips	166	0.234 (0.254)	0.271 (0.262)	0.252 (0.192)	0.206 (0.234)	0.168 (0.231)	0.198 (0.205)	0.036	0.2347	<.0001	0.0039
Shopping, mean (SD), proportion of trips	166	0.174 (0.211)	0.189 (0.188)	0.179 (0.158)	0.158 (0.208)	0.198 (0.266)	0.192 (0.205)	0.4871			
Tourism, mean (SD), proportion of trips	166	0.013 (0.085)	0.022 (0.093)	0.017 (0.040)	0.018 (0.073)	0.023 (0.127)	0.029 (0.098)	0.7565			
Worship, mean (SD), proportion of trips	166	0.094 (0.194)	0.084 (0.143)	0.087 (0.133)	0.099 (0.182)	0.047 (0.161)	0.049 (0.111)	0.0105	0.7307	0.0034	0.0002

Data from 2020 alone showed that pandemic-related change in any driving across the three epochs differed by gender (*p* = .019), race (*p* = .005), and reported stress due to social distancing (p = .017). During the Early part of 2020, the proportion of days with any driving was the same for men and women (0.740 ± 0.042 vs. 0.708 ± 0.035, *p* = .44), but men drove on a greater percentage of days than women during the Mid (0.500±0.047 vs. 0.359 ± 0.038, *p* = .002) and Late (0.542 ± 0.049 vs. 0.425 ± 0.041, *p* = .02) periods of the year. African Americans and Whites had similar proportions of days with any driving during the Early (0.733±0.058 vs. 0.715±0.025, *p* = .77) and Mid (0.406±0.064 vs. 0.452±0.028, *p* = .49) portions of the year, but Whites had a higher proportion of days driving during the later part of the year compared to African Americans (0.572±0.029 vs. 0.395±0.068, *p*=.01). There was no association of stress due to social distancing and proportion of days driving at all during the Early (r = −0.0341, *p* = .64) and Mid (r = 0.037, *p* = .62) portions of the 2020, but increasing reported stress during the later portion of the year was associated with increasing proportion of days with any driving (r = 0.142, *p* = .05).

Belief in risk of contracting COVID-19 interacted with time (*p*=.006). Persons who answered Unsure to the survey item asking whether they believed that they were at risk of being infected with COVID-19 showed more overspeeding (0.018±0.004) than those who answered Yes (0.001 ± 0.003, *p*=.002) or No (0.003 ± 0.004, *p*=.02) during Early 2020, but there was no difference among the groups for Mid and Late portions of the year.

The pandemic was associated with decline in the proportion of trips made to places of worship and restaurants, but an increase in the proportion of trips made to obtain food (Table 2). Age was significantly associated with change in trips for worship (*p* = .03). The form of this interaction was that the absolute value of the Pearson correlation coefficient (Fisher’s Z transformation) of age with proportion of trips for worship changed direction from very positive in the Early (r=+0.03, 95%CI=−+0.13 to +0.18) and Mid (r=+0.06, 95%CI=−0.10 to +0.21) parts of the year to negative later in the year (r=−0.13, 95%CI=−0.27 to +0.03), though the correlations themselves were not statistically significant within each time epoch. Social distancing stress scores interacted with time in their association with proportion of trips for (*p* = .02), such that increasing stress due to social distancing was related to smaller proportions of trips for food during the mid-portion of the year (r=−0.14, 95%CI=−0.29 to +0.01, *p* = .07), but there was no association during the Early (r=+0.03, 95%CI=−0.13 to +0.18, *p*=.71) or Late (r=−0.002, 95%CI=−0.002 to +0.15, *p* = .98) periods in the year.

## Discussion

Although emerging research is reporting age differences between younger and older adults in reactions to the COVID-19 pandemic, little research to date has explored the multidimensional impact of the COVID-19 pandemic on older populations. Results showed that among older adults, who are at higher risk for developing severe complications and death from COVID-19, individual differences in demographic factors, beliefs, and feelings are associated with pandemic-related behaviors and behavior changes.

### Changes in Driving Behaviors Among Older Adults

Regardless of gender, race and socioeconomic status, during and after the rapid acceleration of US COVID-19 cases in the spring of 2020 (Figure 1), older adults became less likely to drive at all, took fewer and shorter trips, and drove slower than before the pandemic. Although the decline in driving space was consistent with findings from studies on younger populations, the reduction in average driving speed was not. In fact, no study, to date, has investigated the changes in driving behaviors of older adults during the pandemic and the few studies that were conducted on the general population have shown an increase in driving speed during the pandemic. A potential explanation for this difference could be that older drivers generally adopt a more cautious style of driving and tend to drive slower, and the pandemic has amplified that style of driving (Doroudgar et al., 2017).

**Figure 2.**
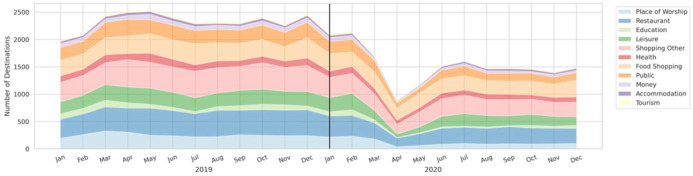
Changes in driving behaviors during and after the rapid acceleration of US COVID-19 cases. Average monthly destinations mapped to one of the 11 identified categories place of worship, restaurant, education, leisure, shopping other, health, food shopping, public, money, accommodation, and tourism.

Furthermore, in mid and late 2020, a significantly smaller portion of older adults’ trips was made to restaurants or places of worship, compared to the same time in 2019 (Figure 2). This is unsurprising because restaurants and places of worship may have been shut down. However, since there was no coordinated, national response to the pandemic in the US regarding school and business closings, we were unable to determine clear timelines for when sentinel events such as business and school closings and openings occurred. Additionally, compared to 2019, a significantly greater portion of older adults’ trips was for food shopping during the pandemic. This finding can be explained by the fact that older adults not only made fewer trips overall but also these outings were more likely to be for necessities.

Pandemic-related protective actions, beliefs, and feelings among older adults

An important protective action against the virus, mask-wearing, was more likely to be reported by persons with one or more medical risk factors for COVID-19 (e.g., asthma and chronic heart condition). This is in accordance with the “perceived susceptibility” construct of the Health Belief Model, which states that an increase in perceived susceptibility to a health problem increases engagement in protective behaviors that can help reduce the risk of developing that problem (Champion & Skinner, 2008). In addition, a nationally representative sample of adults of all ages found that persons with some COVID-19 medical risk factors, those with allergies and compromised immune systems were more likely to wear masks than those without the condition, but persons with other medical risk factors (e.g., asthma and high blood pressure) were not (Yek et al., 2022). Due to the small number of persons with most individual medical risk factors, we were unable to compare across individual conditions in this study.

An important theme arising from this research is regarding the stress due to social distancing. In fact, older adults who experienced more stress in response to recommendations for social distancing were likely to display a smaller decrease in driving. Speculatively, the relationship between driving and stress may be because participants who had smaller decreases in driving were those who had to drive during the pandemic regardless of whether they wanted to or not (e.g., essential workers). That is, participants who drove more and visited more places may have had more interactions with others, increasing their perceived likelihood of contracting COVID-19, causing them additional stress. Additionally, the findings show that older adults with more stress were also more likely to display negative adaptive behaviors. Generally, people participate in positive or negative coping strategies in order to reduce stress. Persons who live alone appeared to be especially vulnerable to adverse effects of the pandemic, in that they were less likely to protect themselves by wearing masks, and more likely to engage in maladaptive behaviors. Individuals living in zip codes with higher social deprivation had more negative coping behaviors. The results highlight the need to further study how public health guidelines and recommendations can be outlined and advertised to reduce stress and promote positive coping strategies among older adults.

Another important theme in this research is regarding older adults’ beliefs of their risk of COVID-19. Older adults who believed that they could protect themselves from COVID-19 were more likely to act in accordance with those beliefs, showing higher adherence to mask-wearing and engagement in cleaning activities. Although not surprising, these results emphasize the danger of misinformation (e.g., “Masks don’t work”) to engaging in appropriate protective behaviors (Hall et al., 2021; Su, 2021). This theme also aligns with the “perceived severity” construct of the Health Belief Model; that is, a higher perceived severity can lead to a higher likelihood of engagement in health-promoting behaviors (Champion & Skinner, 2008).

Finally, living alone and lower education were among other social determinants that accounted for some pandemic-related behavior changes. In fact, older adults who lived alone and those who had fewer years of education were less likely to wear masks and took part in more maladaptive behaviors. This is in line with previous studies showing that older adults living alone and with lower education level are more susceptible to negative changes during the COVID-19 pandemic (Hawkins et al., 2020; Lehtisalo et al., 2021). Considering the profile of these individuals, they should be one of the primary targets of prevention and treatment interventions in response to the effects of the pandemic.

### Gender and Race Differences in Pandemic-Related Behaviors

Following the onset of COVID-19, older adults who were women became differentially more likely to engage in cleaning (e.g., cleaning door handles and washings hands after being in public) and positive coping behaviors (e.g., exercising and taking breaks from the news), compared to men. They also reduced their number of days of driving more than men. These results can be, at least partly, explained by the fact that even before the COVID-19 pandemic, compared to older men, older women had a smaller life-space and were more likely to do cleaning activities (Rosso, Grubesic, Auchincloss, Tabb, & Michael, 2013Rosso et al., 2013). Additionally, the results are in accordance with previous findings that the life-threatening COVID-19 pandemic situation has not alarmed older men as much as older women (Perracini et al., 2021). These findings suggest that gender-related behaviors may be implicated in COVID-19 vulnerability; thus, it is important to leverage sociocultural norms pertaining to gender roles to design effective interventions for the entire population in response to the pandemic.

Furthermore, compared to older adults who were White, African Americans were more likely to wear masks, engage in cleaning activities and exhibit positive coping behaviors. Other research among persons of all ages also indicates that African-Americans^14^ are more likely to exhibit some of these COVID-19 prevention strategies such as mask waring. Additionally, African Americans also had a lower proportion of days driving during the later part of the year, which is in line with findings from before the COVID-19 pandemic showing African Americans face a greater risk of driving decline compared to whites (Babulal et al., 2020). A potential explanation for this trend might be that because African Americans experience a disproportionate burden in morbidity and mortality from the COVID-19 virus, (Andrasfay & Goldman, 2021) they are especially aware of the disabling and deadly effects of COVID-19 and are therefore more likely to take precautionary measures such as masking and cleaning. Alternatively, African Americans may have been more likely to clean and have more positive coping behaviors than Whites even before the pandemic. However, these conjectural explanations should be taken with caution due to the smaller percentage of African American participants in the sample (10.8%).

## Supplemental Material

sj-docx-1-jag-10.1177_07334648221093851 – Supplemental material for Driving, Social Distancing, Protective, and Coping Behaviors of Older Adults Before and During COVID-19 Click here for additional data file. Supplemental material, sj-docx-1-jag-10.1177_07334648221093851 for Driving, Social Distancing, Protective, and Coping Behaviors of Older Adults Before and During COVID-19 by Catherine M. Roe, Sayeh Bayet, Jamie Hicks, Ann M Johnson, Samantha Murphy, Jason M. Doherty and Ganesh M. Babulal in Journal of Applied Gerontology
