# Supplementary material for: Driving, Social Distancing, Protective, and Coping Behaviors of Older Adults Before and During COVID-19
Source: J Appl Gerontol. 2022 May 11;41(8):1831–42. doi: 10.1177/07334648221093851 (PMC9364235; doi:10.1177/07334648221093851)
Supplement: sj-docx-1-jag-10.1177_07334648221093851 – Supplemental material for Driving, Social Distancing, Protective, and Coping Behaviors of Older Adults Before and During COVID-19 [file sj-docx-1-jag-10.1177_07334648221093851.docx]

**Supplementary Data 1.** Subscales created from COVID-19 survey responses. Unless otherwise noted, each item is answered Yes or No.

**Social Distancing**

During the COVID-19 pandemic, have you:

1. Gone out to a restaurant, bar, club, or other place where people gather?
2. Visited with older friends, relatives or neighbors? Older = 60 years old or older
3. Gone to the grocery store or pharmacy?
4. Gone to a friend, neighbor or relative's house (that is not your own)?
5. Had more than 10 friends, neighbors, or relatives over to your house?
6. Gone to a family gathering where there were more than 10 people such as a reunion, wedding, funeral, or birthday party?
7. Gone to a gathering of friends where there were more than 10 people such as a party, wedding or concert?
8. Gone to a faith based gathering such as a church, synagogue, temple, or mosque?

**Masking**

1. Did you wear a mask or face covering (e.g., bandana, scarf, pieces of fabric, etc.) at the restaurant, bar, club, or other place where people gather?
2. Did you wear a mask or face covering (e.g., bandana, scarf, pieces of fabric, etc.) when you visited with older friends, relatives or neighbors?
3. Did you wear a mask or face covering (e.g., bandana, scarf, pieces of fabric, etc.) when you went to the grocery store or pharmacy?
4. Did you wear a mask or face covering (e.g., bandana, scarf, pieces of fabric, etc.) when you went to your friend, neighbor or relative's house (that is not your own)?
5. Did you wear a mask or face covering (e.g., bandana, scarf, pieces of fabric, etc.) when you had more than 10 friends, neighbors, or relatives over to your house?
6. Did you wear a mask or face covering (e.g., bandana, scarf, pieces of fabric, etc.) when you attended the family gathering where there were more than 10 people such as a reunion, wedding, funeral, or birthday party?
7. Did you wear a mask or face covering (e.g., bandana, scarf, pieces of fabric, etc.) when you went to a gathering of friends where there were more than 10 people such as a party, wedding or concert?
8. Did you wear a mask or face covering (e.g., bandana, scarf, pieces of fabric, etc.) when you went to a faith based gathering such as a church, synagogue, temple, or mosque?

**Cleaning**

In the past 7 days, have you (please check all that apply):

1. Cleaned high touch surfaces in the home like door handles, faucets, and remote controls?
2. Cleaned high touched electronics like your smart phone, computer, tablet, or laptop?
3. Washed hands after being in public?
4. Used alcohol-based hand sanitizer?

**Positive Coping**

To cope with social distancing and isolation, are you doing any of the following? (please check all that apply)

1. Taking breaks from watching, reading, or listening to news stories, including social media
2. Taking care of your body, such as taking deep breaths, stretching, or meditating
3. Engaging in healthy behaviors like trying to eat healthy, well-balanced meals, exercising regularly, getting plenty of sleep, or avoiding alcohol and drugs
4. Making time to relax
5. Connecting with others, including talking with people you trust about your concerns and how you are feeling
6. Contacting a healthcare provider

**Negative Coping**

To cope with social distancing and isolation, are you doing any of the following? (please check all that apply)

1. Smoking more cigarettes or vaping more
2. Drinking alcohol
3. Using prescription drugs (like Valium, etc)
4. Using non-prescription drugs
5. Using cannabis or marijuana
6. Eating high fat or sugary foods
7. Cutting or self-injury
8. Over exercise 0.00%
9. Eating more food than usual
10. Eating less food than usual

**Maladaptive Behaviors**

In the past week, have the following behaviors increased in your household (please check all that apply):

1. Interpersonal conflict with family members or loved ones
2. Snapping at or yelling at family members
3. Corporeal punishment of children
4. Corporeal punishment of pets
5. Interpersonal conflict with friends or coworkers

**Supplementary Table 1**. The mapping between place types and destinations that was used to categorize the destinations.

|  | **Place Type** | **Point Of Interest Examples** |
| --- | --- | --- |
| **1** | Place of Worship | Church, mosque, synagogue |
| **2** | Restaurant | Cafe, bar, restaurant, food court |
| **3** | Education | School, university |
| **4** | Leisure | Theatre, cinema, park, stadium |
| **5** | Shopping other | Florist, bookshop, mall, department store |
| **6** | Health | Pharmacy, hospital, doctors, dentist |
| **7** | Food shopping | Supermarket, bakery, convenience store |
| **8** | Public | Police, courthouse, |
| **9** | Money | Bank, ATM |
| **10** | Accommodation | Hotel, motel, hostel |
| **11** | Tourism | Museum, attraction, memorial |
